# Duration of annual canine flea and tick protection provided by dog owners in Spain

**DOI:** 10.1186/s13071-018-3043-x

**Published:** 2018-08-07

**Authors:** Robert Lavan, Rob Armstrong, Federica Burgio, Kaan Tunceli

**Affiliations:** 1 Outcomes Research, MSD Animal Health, Center for Observational and Real-World Evidence, Merck & Co., Inc., Kenilworth, NJ USA; 2 MSD Animal Health, 2 Giralda Farms, Madison, NJ USA; 3 MSD Animal Health, Polígono Industrial El Montalvo. C/ Zeppelin, 6 - Parcela 38, 37008 Carbajosa de La Sagrada, Salamanca, España

**Keywords:** Ectoparasiticide, Dog, Veterinary practice, Duration, Flea, Tick

## Abstract

**Background:**

Doses of flea and tick medication acquired by dog owners over a 12 month period were determined from veterinary hospital transaction records in Spain. The number of months of flea and tick protection potentially obtained by dog owners prescribed fluralaner, a flea and tick medication with a 12 week re-dosing interval, was compared with months of flea and tick protection obtained by dog owners prescribed monthly oral or spot-on products. Prior studies in human and veterinary medicine have suggested that longer-acting medications benefit patients by providing improved adherence to provider recommendations.

**Results:**

Dog owners took home, on average, significantly more months of protection when they obtained the 12 week duration product fluralaner (4.3 months) than they did when they obtained other flea and tick products providing 1 month of protection [3.24 months (oral), 2.9 months (spot-on)]. Many dog owners (46–64%) obtained only one dose of flea and tick product each year, regardless of the duration of protection offered by the product. Significantly more dog owners obtained 7–12 months of protection when they were prescribed fluralaner (15.7%) by their veterinarians compared with dog owners prescribed monthly flea and tick products [6.8% (oral), 8.3% (spot-on)].

**Conclusion:**

Veterinary prescription of fluralaner delivers more months of potential flea and tick protection as shown by dog owner acquisition of flea and tick medication. The use of a longer-acting medication requires the administration of fewer doses and may translate into better adherence to veterinary ectoparasite control recommendations.

## Background

Spain has a range of ectoparasites that parasitize pets and potentially the family they live with. Several species of fleas and ticks are established across its various bioclimatic zones [1, 2]. These parasites are a potential threat all year and this provides a constant challenge for veterinarians and pet owners. Protecting pets from adverse effects of ectoparasites, including blood loss, ectoparasite-induced skin diseases, and vector transmitted pathogens could require a long term protection coverage for ticks and fleas [3–5].

A cross-sectional spatial survey from Spain, carried out from May 2013 to mid-2015, identified *Ctenocephalides felis*, *Ctenocephalides canis* and *Pulex irritans* as the main flea species infesting dogs (total of 3031 fleas). All three species were detected all year, although *C. felis* represented the most abundant (81.7%) and widely distributed species [1]. A tick survey carried out in Spain demonstrated that *Rhipicephalus sanguineus* adult ticks were active throughout the year, with a peak prevalence from March to July. The other tick species were also collected throughout the year, with an autumn-winter peak for *Dermacentor reticulatus*, but without clear seasonality for *Ixodes* species [2]. Following recommendations from the European Scientific Counsel Companion Animal Parasites (ESCCAP), ectoparasite prophylaxis should cover the parasites complete activity period [6]. There is a need for a long-lasting tick and flea protection, and not only during spring or summer. Considering errors in the application of these products that can reduce their effectiveness [7], veterinarians should not only prescribe a medication prophylaxis adequate to the territory ectoparasite pressure but should also explain all aspects related to effectiveness to improve owner compliance. Therefore, for a dog owner to adhere to veterinary recommendations it is necessary to acquire and correctly deliver sufficient product doses to the dog for up to 12 months of flea and tick protection. Many monthly oral or monthly spot-on flea/tick medications are packaged with 3, 6 or 12 doses, to encourage dog owners to acquire enough doses for extended protection.

Dog owners in Spain have many choices of ectoparasiticide for treating or protecting their dogs from flea and tick infestation. Many of these products are topically applied spot-ons with a labelled re-treatment interval of one month. There are also orally administered products that have a one month recommended re-treatment interval. Most spot-on and a few oral products can be obtained without a prescription outside the veterinary hospital. The isoxazoline class products are a newer type of oral flea and tick medication that require a veterinary prescription for administration to dogs in Spain, therefore the veterinarian plays an active role in guiding the attitude and actions of the owner.

Fluralaner (BRAVECTO®, MSD Animal Health, Giralda Farms, NJ, USA) is an isoxazoline class compound with an extended 12 week re-treatment interval that was introduced for dogs in Spain in April, 2014. Clinical trials demonstrated a high level of efficacy against fleas and ticks on dogs throughout the re-treatment interval [8, 9]. This product kills ticks and adult fleas and is indicated for the treatment and prevention of flea infestations (*Ctenocephalides felis*) and the treatment and control of tick infestations [*Ixodes ricinus* (black-legged tick), *Dermacentor variabilis* (American dog tick), *Dermacentor reticulatus* and *Rhipicephalus sanguineus* (brown dog tick)] for up to 12 weeks in dogs and puppies 8 weeks of age and older, and/or weighing 2 kg or greater [10].

Medications are only effective if they are taken as prescribed without missed doses. Longer acting medications require the administration of fewer doses and previous research suggests that these might enable patients to better adhere to health provider recommendations. Studies on human patient adherence to prescribed courses of treatment generally report an inverse relationship between dosing frequency and medication adherence, with significantly higher adherence rates reported for medications with a longer duration of action and decreased dosing frequency [11–14]. This inverse link between dosing frequency and adherence has been demonstrated across an array of drug classes including antibiotics, steroids and medications that treat respiratory disease, diabetes mellitus and hypercholesterolemia [15–18]. This relationship is partially responsible for the current trend toward longer-acting formulations in human medicine [17, 18]. A study involving veterinarians and dog owners in the USA suggests that a similar relationship exists between less frequent dosing and increased adherence to veterinary flea/tick medication recommendations [19]. All veterinarians prescribing flea and tick medications should document and report any adverse reaction observed during the treatment period.

The 12 week re-treatment interval for fluralaner provides a convenient prevention and/or treatment option for dog owners because of the less frequent dosing schedule compared to medications which must be re-dosed monthly. A USA study [19] found that dog owners obtained approximately 6 months of protection with fluralaner per year. This represents a longer duration of coverage than previously reported in 2015 market research studies [20, 21] which were completed before the launch of fluralaner. The authors of the USA study have described the need for further research studies to examine if longer re-treatment intervals indeed contribute to improved dog owner adherence with veterinary flea and tick control recommendations. For this purpose, the present study aims to determine whether the reduced dosing frequency of fluralaner is associated with a greater duration of flea and tick protection obtained in comparison to monthly flea and tick medications.

## Methods

This study is a retrospective, observational study of veterinary transactional records from animal hospitals in Spain. The aim of the study was to measure dog owner acquired doses of fluralaner and to compare it with dog owner acquired doses for other flea and tick medications which have a monthly re-treatment interval. The yearly doses of flea and tick medication were compared with known veterinary recommendations for annual protection.

Blinded transactional data for the ectoparasiticidal products were obtained from Spanish veterinary clinics through a proprietary medical records data collection service. Collected data did not contain any proper names or addresses for pet owners or their pets but did contain individual dog age, age block and body size classification. Code numbers concealed owner patient identity while allowing serial transactions to be matched to individual dogs through the study period. The study period extended from April 2014, the first month that fluralaner was sold in Spain, through the end of December, 2016, when the data were downloaded. The study collected transactions from four oral products, including fluralaner and three monthly products, and 19 spot-on products which collectively make up approximately 95% of ectoparasiticidal treatments in Spain. The data sharing agreement required that product brand names not be identified, other than fluralaner. The monthly anti-ectoparasitic products were grouped into the categories of “oral” or “spot-on” products rather than listing them by individual brands. The results from the three monthly oral products and 19 spot-on products were each combined into comparator categories of monthly oral products and monthly spot-on products. These products all treat flea infestations and most also treat tick infestations on dogs; therefore the products are referred to throughout the manuscript as flea/tick products. Insecticidal collars as well as products containing a heartworm medication were excluded from the analysis. However, approximately 1.9% of the dogs in the data received a collar at the same time that these dog owners also received a product for flea and tick protection. It is therefore likely that the owners were acquiring the collar for an indication other than flea and tick protection, *Leishmania* for example, and collars were not included in the duration of protection analysis.

Inclusion and exclusion criteria were applied to the data to remove non-canine species and duplicate entries and to ensure proper dose counts, especially with multi dose packs. An additional criterion was applied to the initial database to ensure that each patient record represented transactions for one dog and not multiple dogs. Dog owners were classified as “pure users” based on at least one full year history of using a single flea/tick medication. All dogs older than 8 weeks were included in the analysis. To eliminate situations where transactions may have been made for more than one dog, canine patients were removed if more than 12 doses were given in a single transaction or more than 24 doses were obtained in 12 months. This is based on the assumption that a dog owner might acquire a maximum of 12 months of product for the first year and then get a second 12 months of product for the following year before the current 12 month period ended. Dog owners were considered to be acquiring doses for multiple dogs when the recorded transactions were in excess of expected maximum doses for a single dog.

The date of the first dog owner transaction in the study period was identified as the Index Date (ID). The ID had to be prior to December 31, 2015 in order to have a full 12 month window for the dog owner to acquire additional doses. The follow-up period was described as the 12 months following the ID. Only transactions for the index drug (i.e. pure user of a flea/tick product) made during the follow-up period were considered in estimating duration of coverage. Each dose of fluralaner is labeled to provide flea/tick protection for 12 weeks, and 4.3 doses provide 12 months of coverage. The other oral and topical products are labeled for monthly dosing and 12 doses provide 12 months of coverage.

Therefore, according to product labels, each fluralaner dose and monthly product dose were considered to provide 84 days (12 weeks × 7 days/week) and 30 days coverage for flea and tick prevention respectively. In aggregating days from each prescribed dose, the potential coverage was determined from the total doses obtained in the one year follow-up period that could be used in the same period. This meant that doses obtained late in the 12 month follow-up period would not count for a full month but would only count for a fraction of the last month. For example, if the first monthly dose was acquired on January 1 and the last dose was acquired the following December 15, the pet would be credited with full doses up to the last dose, which would count for 15/31 days or approximately half of the last month. Doses or proportion of doses that might have provided flea/tick protection after the 12 month period were not included in calculating duration of flea/tick protection. It was assumed that all doses were given on time and consecutively if multiple doses were obtained in one transaction.

Limited canine demographic data were obtained with the transaction data. The classification of body size was included with the downloaded transaction data and are not based on specific size or weight measurements.

Descriptive statistics were calculated for dog age, age block and body size and expressed as frequencies, percentages, means and standard deviations. The average number of months of flea/tick coverage and mean number of acquired doses that were obtained by dog owners were calculated as frequencies, percentages, means and standard deviations, as well. Means were compared across groups using a Chi-square test or t-test with significance set at *P* < 0.05.

## Results

The unfiltered database included 30,738 dogs whose owners received flea/tick products licensed in Spain, between April 2014 and December 2016. The final database, which met all inclusion and exclusion criteria, included 30,020 dogs and their flea/tick transaction records.

The database contained patient demographic data including the dog age, dog age block and body size (Table 1). However, more than half of the dogs did not have any age or body size data recorded. The mean age of those dogs for which data were available for each of the different medication categories ranged from 3.9–4.7 years and most dogs were in the 1–8 year age block. For dogs with a body size description, approximately 56% were classified as “small”, 17% were “medium” and 27% were “large”. Because of the large sample size, the dog age, age block and body size in this study were statistically significantly different across different flea and tick product groups (Dog age: *F*_(2, 3200.6)_ = 43.5, *P* < 0.0001; Age block: *χ*^2^ = 159.59, *df* = 8, *P* < 0.001; Body size: *χ*^2^ = 138.33, *df* = 6, *P* < 0.0001) . However, the small differences were not considered to be clinically meaningful.

**Table 1:** Demographics of the study dog population from veterinary practices in Spain

	Fluralaner	Monthly oral products	Monthly spot-on products	Total
Number of dogs	3483	5969	20,568	30,020
Dog age				
Mean ± SD (years)	4.0 ± 3.7	4.7 ± 3.9	3.9 ± 3.8	4.1 ± 3.8
Dog age block (*n*, %)				
8 weeks to 1.0 year	510 (15)	575 (10)	3244 (16)	4329
1.1–8.0 years	772 (22)	1212 (21)	4132 (20)	6116
8.1–12.0 years	170 (5)	322 (6)	923 (5)	1415
Over 12.1 years	35 (1)	85 (1)	246 (1)	366
Missing data	1996 (57)	3775 (63)	12,023 (59)	17,794
Dog body size (*n*, %)				
Small	849 (24)	1231 (21)	5253 (26)	7333
Medium	287 (8)	404 (7)	1551 (8)	2242
Large	536 (15)	666 (11)	2326 (11)	3528
Missing data	1811 (52)	3668 (61)	11,438 (56)	16,917

*Abbreviations*: n, number of dogs; SD, standard deviation

The average number of doses and the average months of flea and tick protection acquired by pet owners in Spain over the 12 month follow-up period are shown in Table 2. Owners prescribed fluralaner for their dog took home significantly more months of coverage, on average, than pet owners prescribed monthly oral (*t*_(8173)_ = 19.34. *P* < 0.0001) or monthly spot-on (*t*_(24049)_ = 31.42, *P* < 0.0001) flea/tick products (Table 2). This study found that dog owners who took home fluralaner received enough doses to deliver 4.3 months of coverage per year compared to 3.2 months for dog owners who used monthly oral flea/tick products and 2.9 months for dog owners using monthly spot-on flea/tick products. Expressed as a percentage, pet owners who acquired either monthly oral or monthly spot-on products had to obtain, on average, an additional 34% and 50%, respectively, of their actual obtained doses to match the duration of coverage provided by obtained fluralaner doses.

**Table 2:** Mean acquired doses and months of flea/tick coverage in Spain over 12 months

	12 week Fluralaner	Monthly oral products	Monthly spot-on products
Mean doses acquired ± SD*	1.6 ± 0.9^a^	3.2 ± 2.9^b^	2.9 ± 2.5^c^
Mean months of coverage ± SD*	4.3 ± 2.5^a^	3.2 ± 2.9^b^	2.9 ± 2.5^c^

*Different superscript letters indicate statistically significant differences at *P* < 0.05 (fluralaner *versus* monthly oral (*t*_(8173)_ = 19.34. *P* < 0.0001) or monthly spot-on (*t*_(24049)_ = 31.42, *P* < 0.0001)).

**Fig. 1:**
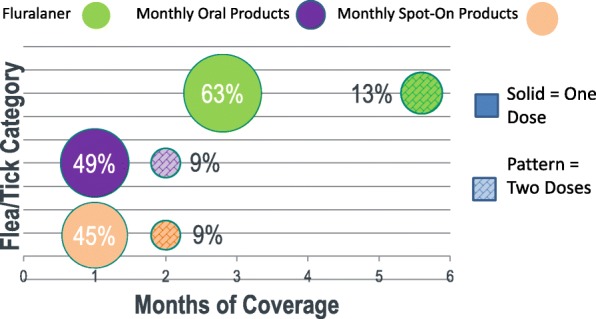
Dog owners who obtain one or two doses of flea/tick medication over a 12 month period

A year of monthly transactions by dog owners of flea and tick products are summarized (Table 3). Most dog owners acquired 1 or 2 doses of flea/tick medication per year (Table 3, Fig. 1). Over the calendar year, 64% of pet owners who received fluralaner received one dose, providing 12 weeks (2.8 months) of flea/tick coverage and 13% received the second dose providing 24 weeks of protection. Approximately 50% and 46% of dog owners acquired only one dose, providing one month of protection through oral and spot-on products, respectively. Approximately 9% of pet owners who acquired the monthly oral or spot-on products also obtained a second dose.

**Table 3:** Months of flea/tick coverage obtained for dogs in Spain in a 12 month period

Months of coverage acquired	Fluralaner (*n* = 3483)	Monthly oral products (*n* = 5969)	Monthly spot-on products (*n* = 20,568)	Total (*n* = 30,020)
	% (*n*)	% (*n*)	% (*n*)	*n*
1.0–1.9	0	49.3 (2940)	46.1 (9485)	12,425
2.0–2.9	63.6 (2214)	9.2 (548)	8.7 (1791)	4553
3.0–3.9	2.2 (75)	7.0 (416)	12.7 (2609)	3100
4.0–4.9	1.6 (54)	2.0 (121)	18.5 (3812)	3987
5.0–5.9	14.9 (518)	1.0 (62)	2.1 (437)	1017
6.0–6.9	2.1 (74)	24.8 (1479)	3.6 (730)	2283
7.0–7.9	1.6 (57)	1.0 (60)	1.1 (217)	334
8.0–8.9	6.3 (220)	0.5 (31)	2.9 (590)	841
9.0–9.9	1.3 (45)	0.4 (22)	1.0 (203)	270
10.0–10.9	2.1 (74)	0.4 (22)	0.6 (121)	217
11.0–11.9	3.7 (129)	0.4 (23)	0.5 (97)	249
12	0.7 (23)	4.1 (245)	2.3% (476)	744

Dog owners who obtained the fluralaner flea/tick product were significantly more likely to obtain 7–12 months of coverage in a year compared with dog owners who obtained the monthly oral (*χ*^2^ = 240.22, *df* = 1, *P* < 0.0001) or monthly spot-on products (Table 4; *χ*^2^ = 233.05, *df* = 1, *P* < 0.0001).

**Table 4:** Dog owner flea/tick annual medication doses for 1–6 months and 7–12 months

Months of coverage acquired*	Fluralaner	Monthly oral products	Monthly spot-on products
1.0–6.0	84.3%^a^	93.3%^b^	91.7%^c^
7.0–12.0	15.7%^a^	6.8%^b^	8.3%^c^

*Different superscript letters indicate statistically significant differences at *P* < 0.05 (1–6 and 7–12 months of coverage in a year, fluralaner *versus* monthly oral (*χ*^2^ = 240.22, *df* = 1, *P* < 0.0001) or monthly spot-on products (*χ*^2^ = 233.05, *df* = 1, *P* < 0.0001)

## Discussion

The objective of this study was to measure the months of flea and tick protection acquired by dog owners in Spain from veterinary hospitals and to examine whether duration of potential flea and tick protection differs between pet owners who were prescribed fluralaner with a 12 week re-treatment interval compared with monthly oral or monthly spot-on flea/tick medications. Based on analysis of transactional data from over 30,000 dogs, dog owners obtained significantly more months of flea and tick protection with the longer acting 12 week fluralaner flea/tick treatment compared with dog owners who acquired either monthly oral flea/tick products or monthly topical flea/tick products. Over a 12 month period, the average Spanish dog owner who was prescribed a monthly oral or monthly spot-on flea/tick medication by their veterinarian received between 2.9–3.2 months of coverage (Table 2). The average dog owner who was prescribed fluralaner for their dog obtained 4.3 months of protection, significantly more months of coverage than average dog owners who were prescribed monthly oral or monthly spot-on flea/tick products. There was a potential 34% gain in months of protection for the 12 week product compared with the monthly oral product and a potential 50% gain in protection *vs* the monthly topical product. This potential increase in flea and tick protection through the use of a product with a longer duration of action is consistent with increased adherence seen with use of longer acting products by human and veterinary patients facing other health conditions that require prolonged dosing. The increased flea/tick protection seen in dogs that are prescribed fluralaner might result from the longer re-treatment interval which is considered to be a more convenient option for dog owners in a recent study [22].

However, these data on the number of flea/tick product doses obtained by dog owners from veterinarians in Spain are not proof that these owners administered all acquired doses. Doses may have been administered as recommended, partially administered or not administered at all. The study measures ectoparasiticide doses acquired through veterinarians and cannot rule out the possibility that other doses were obtained from other sources. This type of transactional analysis is an imperfect estimate of medication use in a given therapeutic area; however, it provides a rough measurement of potential duration of protection of dogs against flea and tick infestation. Other investigational methods, such as asking dog owners to enumerate their actual doses administered, undergo a recall bias if the administered dose was not recent. Pet owner surveys suffer from a self-incrimination bias if pet owners avoid accurately reporting un-administered doses so as not to be seen as a less competent pet owner. However, for the same time period, there are fewer opportunities to forget to re-administer a longer acting treatment than shorter acting treatments. Quarterly deworming, a common strategy recommended by ESCAAP for dogs and cats, is in alignment with fluralaner dosing and might help pet owners better remember 12 week control for ectoparasites [6].

Many dog owners acquired only one dose of flea/tick medication for their dog during the year (Table 3 and Fig. 1). The potential ectoparasite control efficacy attained by administering a single dose is very limited regardless of the time of year when the dose is given. Veterinarians would prefer that these dogs receive flea/tick treatments throughout the time when fleas and ticks present the greatest risk of infesting the dog, and this cannot be achieved by one dose. However, it is possible that dog owners who acquire one dose of flea/tick medication do this to administer the treatment driven by the presence of visible ectoparasites on their dog rather than as a preventative in anticipation of flea/tick season. Recommendations for treating flea infestations focus on providing protection for 90 days and require application of at least two ectoparasiticide doses, and usually more, when the treatment interval is one month [23]. Therefore, control of home flea infestations is completely dependent on sufficient repeated monthly treatment administrations of an effective product at correctly timed intervals. Administration of only one dose of a monthly product without re-treatment will not succeed at controlling fleas and it is a certainty that households giving only one monthly treatment will see their flea population rapidly rebound as the efficacy tapers and as further flea life stages in the household environment mature and re-infest the dog. In contrast, a dog owner administering a single dose of a product like fluralaner that has a 12 week efficacy duration can eliminate the flea life stages in the home environment through the extended efficacy against fleas as they continue to mature [24]. The dog owner will then need to retreat with fluralaner at the end of the 12 weeks to provide protection against re-infestation from outdoor sources or from visiting animals. Recommendations from the European Scientific Counsel Companion Animal Parasites (ESCCAP), indicating that ectoparasite prophylaxis should cover the parasites complete activity period [6]. For a dog owner to adhere to veterinary recommendations it is necessary to acquire sufficient product doses for at least 6 months of flea and tick protection. Very few pet owners in this study who obtained a monthly treatment actually went home with more than 7 months of flea/tick medication (Table 3; oral = 6.8%, spot-on = 8.4%).

Consistent with relatively low estimates of mean duration for protection (range from 4.34 months to 2.89 months, Table 2), most dog owners took home 6 months or less of flea and tick protection in a 12 month period (Table 4). Dog owners who acquired 7–12 months of flea and tick protection for their dog were significantly more likely to take home fluralaner (Table 4). The increase in months of potential coverage for dog owners who obtained fluralaner results from acquiring more potential months of coverage in 12 months than others who have acquired monthly products. Although we may not identify exact reasons/motivations for this increase within the scope of the current study, convenience of an extended release product might motivate pet owners to be more compliant to veterinary recommendations and less frequent re-dosing schedule might provide less opportunity to miss a dose due to forgetfulness or any other reason. The convenience of administering a product that provides 12 weeks of coverage compared with a monthly treatment interval was previously identified as an important factor for treatment satisfaction by 88% of dog owners using a 12 week flea and tick product [22]. The same study also found that 65% of dog owners that were prescribed monthly flea/tick products expected that they were more likely to give the next fluralaner dose on time compared with their expectation for re-dosing a monthly flea/tick product.

By recommending a longer acting flea and tick medication, veterinarians have the potential to improve pet owner adherence and increase the length of ectoparasites protection each year.

A challenge for all flea and tick products is that continued parasite control is completely dependent on owner re-administration of subsequent doses as the end of the treatment interval is reached. For monthly oral and topical products, this time is reached 12 times each year and for fluralaner, this is just over 4 times per year. Prescribing veterinarians are very unlikely to receive accurate and timely updates from dog owners regarding their true pattern of re-administration of prescribed doses over consecutive months. Therefore, in most cases, veterinarians will not know how much flea and tick coverage was actually given. However, each dose of fluralaner delivers a duration of effect that is approximately three times longer than administered with a monthly product and the efficacy of a single dose of fluralaner will not have treatment gaps in a three month period. A small proportion of dog owners in Spain acquired sufficient doses of monthly oral products (7.0%) or monthly spot-on products (12.7%) to match the proportion of dog owners who obtained the 12 week duration delivered by a single dose of fluralaner (63.6%, Table 3). The owners obtaining monthly treatments will still need to administer these products at the correct re-treatment intervals in order to achieve similar parasite control (assuming the products deliver a similar level of efficacy).

Dosing compliance is an important consideration when prescribing any medication that will be dispensed to the dog owner for administration. Therefore, the opportunity to improve compliance through the prescription of longer acting therapeutics is likely to lead to an improved treatment response. Investigations in human medicine have shown that patients prescribed longer acting medications benefit from higher and longer drug levels in serum and show improvements through subsequent reduction in disease clinical signs and better health [11, 13–18].

Further research is needed to investigate whether this extended period of ectoparasite protection translates into improvement in other measures of general health.

## Conclusions

This study demonstrates that owners obtaining a prescription from their veterinarian for a product with 12 weeks duration potentially will protect their dogs against fleas and ticks for a longer period compared to those prescribed products with a monthly duration.

## Abbreviation

ID: Index date, the first date that an ectoparasiticide dose was obtained
